# Learning with repeated-game strategies

**DOI:** 10.3389/fnins.2014.00212

**Published:** 2014-07-30

**Authors:** Christos A. Ioannou, Julian Romero

**Affiliations:** ^1^Department of Economics, University of SouthamptonSouthampton, UK; ^2^Department of Economics, Krannert School of Management, Purdue UniversityLafayette, IN, USA

**Keywords:** adaptive models, experience weighted attraction model, finite automata

## Abstract

We use the self-tuning Experience Weighted Attraction model with repeated-game strategies as a computer testbed to examine the relative frequency, speed of convergence and progression of a set of repeated-game strategies in four symmetric 2 × 2 games: Prisoner's Dilemma, Battle of the Sexes, Stag-Hunt, and Chicken. In the Prisoner's Dilemma game, we find that the strategy with the most occurrences is the “Grim-Trigger.” In the Battle of the Sexes game, a cooperative pair that alternates between the two pure-strategy Nash equilibria emerges as the one with the most occurrences. In the Stag-Hunt and Chicken games, the “Win-Stay, Lose-Shift” and “Grim-Trigger” strategies are the ones with the most occurrences. Overall, the pairs that converged quickly ended up at the cooperative outcomes, whereas the ones that were extremely slow to reach convergence ended up at non-cooperative outcomes.

## 1. Introduction

Robert Axelrod pioneered the area of computational simulations with the tournaments in which game-playing algorithms were submitted to determine the best strategy in the repeated Prisoner's Dilemma game (Axelrod, 1984). Axelrod and Dion (1988) went on to model the evolutionary process of the repeated Prisoner's Dilemma game with a genetic algorithm (Holland, 1975). The genetic algorithm is an adaptive learning routine that combines survival of the fittest with a structured information exchange that emulates some of the innovative flair of human search. Other adaptive learning paradigms are derivatives of either belief-based models or reinforcement-based models. Belief-based models operate on the premise that players keep track of the history of play and form beliefs about other players' behavior based on past observation. Players then choose a strategy that maximizes the expected payoff given the beliefs they formed. Reinforcement-based models operate according to the “law of effect,” which was formulated in the doctoral dissertation of Thorndike (1898). In principle, reinforcement learning assumes that a strategy is “reinforced” by the payoff it earned and that the propensity to choose a strategy depends, in some way, on its stock of reinforcement. On the other hand, Camerer and Ho (1999) introduced in their seminal study a truly hybridized workhorse of adaptive learning, the Experience Weighted Attraction (EWA) model. Despite its originality in combining elements from both belief-based and reinforcement-based models, EWA was criticized for carrying “too” many free parameters. Responding to the criticism, Ho et al. (2007) replaced some of the free parameters with functions that self-tune, while other parameters were fixed at plausible values. Appropriately labeled, the self-tuning EWA, the model does exceptionally well in predicting subjects' behavior in a multitude of games, yet has been noticeably constrained by its inability to accommodate repeated-game strategies. As Camerer and Ho (1999) acknowledge in their conclusion, the model will have to be upgraded to cope with repeated-game strategies “because stage-game strategies (actions) are not always the most natural candidates for the strategies that players learn about” (p. 871)^1^.

In Ioannou and Romero (2014), we propose a methodology that is generalizable to a broad class of repeated games to facilitate operability of adaptive learning models with repeated-game strategies. The methodology consists of (1) a generalized repeated-game strategy space, (2) a mapping between histories and repeated-game beliefs, and (3) asynchronous updating of repeated-game strategies. The first step in operationalizing the proposed methodology is to use generalizable rules, which require a relatively small repeated-game strategy set but may implicitly encompass a much larger space (see, for instance, Stahl's rule learning in Stahl, 1996, 1999; Stahl and Haruvy, 2012). The second step applies a fitness function to establish a mapping between histories and repeated-game beliefs. Our approach solves the inference problem of going from histories to beliefs about opponents' strategies in a manner consistent with belief learning ^2^. The third step accommodates asynchronous updating of repeated-game strategies. The methodology is implemented by building on three proven action-learning models: a self-tuning Experience Weighted Attraction model (Ho et al., 2007), a γ-Weighted Beliefs model (Cheung and Friedman, 1997), and an Inertia, Sampling and Weighting model (Erev et al., 2010). The models' predictions with repeated-game strategies are validated with data from experiments with human subjects in four symmetric 2 × 2 games: Prisoner's Dilemma, Battle of the Sexes, Stag-Hunt, and Chicken. The goodness-of-fit results indicate that the models with repeated-game strategies approximate subjects' behavior substantially better than their respective models with action learning. The model with repeated-game strategies that performs the best is the self-tuning EWA model, which captures significantly well the prevalent outcomes in the experimental data across the four games.

In this study, our goal is to use the self-tuning EWA model with repeated game strategies as a computer testbed to examine the relative frequency, speed of convergence and progression of a set of repeated-game strategies in the four aforementioned symmetric 2 × 2 games. Learning with repeated-game strategies is important on many levels (henceforth, for brevity, we refer to repeated-game strategies as *strategies*, unless there is a risk of confusion). First, identifying empirically relevant strategies can help future theoretical work to identify refinements or conditions that lead to these strategies. The literature on repeated games has made little progress toward this target thus far. Second, pursuing an understanding of the strategies that emerge may also help identify in which environments cooperation is more likely to be sustained. Third, identifying the set of strategies used to support cooperation can provide a tighter test of the theory. For instance, we could test whether the strategies that emerge coincide with the ones that the theory predicts.

Similar to Ioannou and Romero (2014), in the computational simulations, we chose to limit the number of potential strategies considered so as to reflect elements of bounded rationality and complexity as envisioned by Simon (1947). Thus, the players' strategies are implemented by a type of finite automaton called a *Moore machine* (Moore, 1956). According to the thought experiment, a fixed pair of players is to play an infinitely-repeated game with *perfect monitoring* and *complete information*. A player is required to choose a strategy out of a candidate set consisting of one-state and two-state automata. The strategy choice is based on the attraction of the strategy. Initially, each of the strategies in a player's candidate set has an equal attraction and hence an equal probability of being selected. The attractions are updated periodically as the payoffs resulting from strategy choices are observed. The new strategy is chosen on the basis of the updated attractions. Over the course of this process, some strategies decline in use, while others are used with greater frequency. The process continues until convergence to a limiting distribution is approximated.

In the Prisoner's Dilemma game, we find that the strategy with the most occurrences was the “Grim-Trigger.” Moreover, the pairs that converged quickly ended up at the cooperative outcome, whereas the ones that were extremely slow to reach convergence ended up at the defecting outcome. In the Battle of the Sexes game, a cooperative pair that alternates between the two pure-strategy Nash equilibria emerged as the one with the most occurrences. The pairs that alternated were quicker to reach convergence compared to the ones that ended up at one of the two pure-strategy Nash equilibria. In the Stag-Hunt and Chicken games, the “Win-Stay, Lose-Shift” and “Grim-Trigger” strategies were the ones with the most occurrences. Similar to the other games, the automaton pairs that converged quickly ended up at the cooperative outcomes (i.e., the payoff-dominant equilibrium in the Stag-Hunt game, and the conciliation outcome in the Chicken game), whereas the ones that were slow to reach convergence ended up at non-cooperative outcomes.

## 2. The self-tuning EWA with repeated-game strategies

### 2.1. Preliminaries

To simplify exposition, we start with some notation. The stage game is represented in standard strategic (normal) form. The set of players is denoted by *I* = {1, …, *n*}. Each player *i* ∈ *I* has an *action set* denoted by 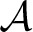
_*i*_. An *action profile a* = (*a_i_*, *a*_−*i*_) consists of the action of player *i* and the actions of the other players, denoted by *a*_−*i*_ = (*a*_1_, …, *a*_*i*−1_, *a*_*i*+1_, …, *a_n_*) ∈ 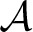
_−*i*_. In addition, each player *i* has a real-valued, stage-game, payoff function *g*_*i*_: 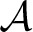 → ℝ, which maps every action profile *a* ∈ 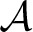 into a payoff for *i*, where 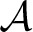 denotes the cartesian product of the action spaces 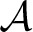
_*i*_, written as 
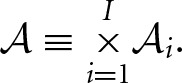
 In the infinitely-repeated game with *perfect monitoring*, the stage game in each time period *t* = 0, 1, … is played with the action profile chosen in period *t* publicly observed at the end of that period. The *history* of play at time *t* is denoted by *h^t^* = (*a*^0^, …, *a*^*t*−1^) ∈ 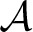
^*t*^, where *a^r^* = (*a*^*r*^_1_, …, *a^r^_n_*) denotes the actions taken in period *r*. The set of histories is given by

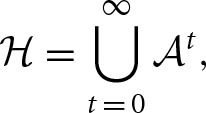



where we define the initial history to the null set 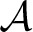
^0^ = {∅}. A *strategy s_i_* ∈ *S_i_* for player *i* is, then, a function 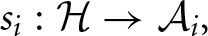 where the strategy space of *i* consists of *K_i_* discrete strategies; that is, *S_i_* = {*s*^1^_*i*_, *s*^2^_*i*_, …, *s^K_i_^_i_*}. Furthermore, denote a strategy combination of the *n* players except *i* by *s*_−*i*_ = (*s*_1_, …, *s*_*i* − 1_, *s*_*i* + 1_, …, *s_n_*). The set of joint-strategy profiles is denoted by *S* = *S*_1_ × … × *S_n_*. Each player *i* has a payoff function π*^t^_i_*: *S* → ℝ, which represents the average payoff per period when the joint-strategy profile is played for *t* periods.

### 2.2. Evolution of learning

Players have attractions, or propensities, associated with each of their strategies, and these attractions determine the probabilities with which strategies are chosen when players experiment. Initially, all strategies have an equal attraction and hence an equal probability of being chosen. The learning process evolves through the strategies' attractions that are periodically updated. Similar to its predecessors, the self-tuning EWA model consists of two variables that are updated once an agent switches strategies. The first variable is *N_i_*(χ), which is interpreted as the number of observation-equivalents of past experience in block χ of player *i*^3^. The second variable, denoted as *A^j^_i_*(χ), indicates player *i*'s attraction to strategy *j after* the χth block of periods. The variables *N_i_*(χ) and *A^j^_i_*(χ) begin with some prior values, *N_i_*(0) and *A^j^_i_*(0). These prior values can be thought of as reflecting pre-game experience, either due to learning transferred from different games or due to pre-play analysis. In addition, we use an indicator function 𝕀(*x, y*) that equals 1 if *x* = *y* and 0 otherwise. The evolution of learning over the χth block with χ ≥ 1 is governed by the following rules:

(1) $$N_{i}\left(\chi \right)=\phi_{i}(\chi )\cdot N_{i}\left(\chi -1\right)+1,$$

and

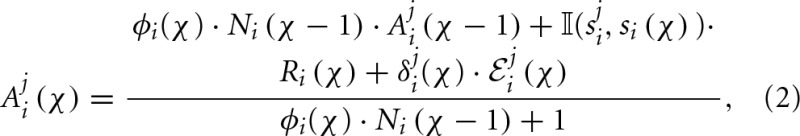



where *R_i_*(χ) is the reinforcement payoff and 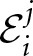
(χ) is the expected forgone payoff to player *i* for strategy *j*.

The reinforcement payoff, *R_i_*(χ), is defined as the average payoff obtained by player *i* over the χth block,

$$R_{i}\left(\chi \right)=\frac{1}{T_{i}(\chi )}\sum_{a\;\in \;h(\chi )}g_{i}\left(a\right),$$

where *h*(χ) is the sequence of action profiles played in the χth block, and *T_i_*(χ)is the χth block's length for player *i*. In addition, the forgone payoffs in the self-tuning EWA model with repeated-game strategies are not as simple as in the case of the self-tuning EWA model with actions, where the opponent's action is publicly observed in each period. To calculate the forgone payoff 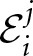
(χ) players need to form beliefs about the current repeated-game strategy of their opponent. In particular, the expected forgone payoff for player *i* of repeated-game strategy *j* over the χth block is the payoff player *i* would have earned had he chosen some other repeated-game strategy *j* given his beliefs about player −*i*'s current repeated-game strategy.

We indicate next how beliefs are specified. To determine the beliefs, let *h*(*t*_1_, *t*_2_) = (*a*^*t*_1_^, *a*^*t*_1_+1^, …, *a*^*t*_2_^) for *t*_1_ ≤ *t*_2_ be the truncated history between periods *t*_1_ and *t*_2_ (all inclusive). Also, let *h* (*t*, *t* − 1) = ∅ be the empty history. Let 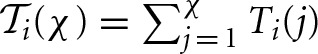 be the total number of periods at the end of block χ. Then, repeated-game strategy *s*_−*i*_ is consistent with 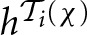 for the last *t*′ periods if

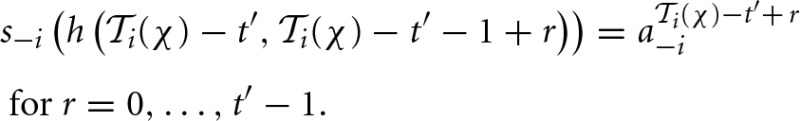



Define the fitness function 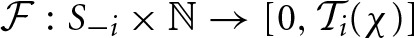 as


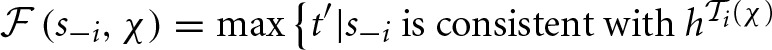
 for the last *t*′ periods}.^4^                 (3)

Define the belief function 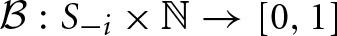 as

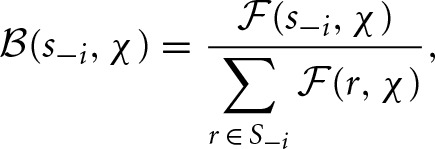



which can be interpreted as player *i*'s belief that the other player was using repeated-game strategy *s*_−*i*_ at the end of block χ. Therefore, the expected foregone payoff for player *i* of strategy *j* over the χth block is given by

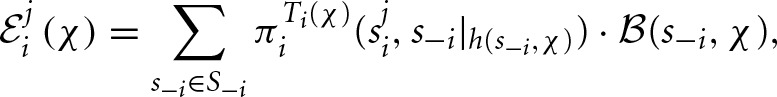



where *s*_−*i*|*h*_ is the continuation strategy induced by history *h* and

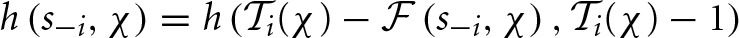



is the longest history such that *s_−i_* is consistent with 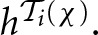


In the original EWA model of Camerer and Ho (1999), the attraction function consisted of the exogenous parameters δ and ϕ. In the self-tuning EWA model, these exogenous parameters were changed to self-tuning functions δ (·) and ϕ (·), referred to as the attention function and the decay-rate function, respectively. The attention function δ(·) determines the weight placed on forgone payoffs. The idea is that players are more likely to focus on strategies that would have given them a higher payoff than the strategy actually played. This property is represented by the following function:

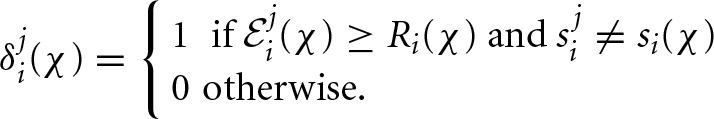



Thus, the attention function enables player *i* to reinforce only *unchosen* strategies with weakly better payoffs. On the other hand, the decay rate function ϕ (·) weighs lagged attractions. When a player senses that the other player is changing behavior, a self-tuning ϕ_*i*_(·) decreases so as to allocate less weight to the distant past. The core of the ϕ_*i*_(·) is a “surprise index,” which indicates the difference between the other player's most recent strategy and the strategies he chose in the previous blocks. First, define the averaged belief function σ : *S*_−*i*_ × ℕ → [0, 1],

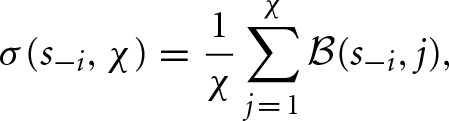



which averages the beliefs, over the χ blocks, that the other player chose strategy *s*_−*i*_. The surprise index 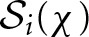 simply sums up the squared deviations between each averaged belief σ(*s*_−*i*_, χ) and the immediate belief 
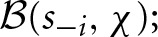
 that is,

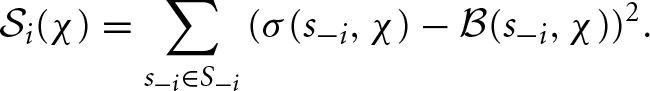



Thus, the surprise index captures the degree of change of the most recent beliefs from the historical average of beliefs. Note that it varies from zero (when there is belief persistence) to two (when a player is certain that the opponent just switched to a new strategy after playing a specific strategy from the beginning). The change-detecting decay rate of the χth block is then

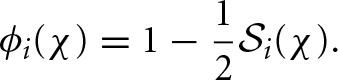



Therefore, when player *i*'s beliefs are not changing, ϕ_*i*_(χ) = 1; that is, the player weighs previous attractions fully. Alternatively, when player *i*'s beliefs are changing, then ϕ_*i*_(χ) = 0; that is, the player puts no weight on previous attractions.

Attractions determine probabilities of choosing strategies. We use the logit specification to calculate the choice probability of strategy *j*. Thus, the probability of a player *i* choosing strategy *j* when he updates his strategy at the beginning of block χ + 1 is

$${\mathbb{P}}_{i}^{\;j}(\chi +1)=\frac{e^{\lambda \cdot A_{i}^{j}(\chi )}}{\sum_{k}^{K}e^{\lambda \;\cdot \;A_{i}^{k}(\chi )}}.$$

The parameter λ ≥ 0 measures the sensitivity of players to attractions. Thus, if λ = 0, all strategies are equally likely to be chosen regardless of their attractions. As λ increases, strategies with higher attractions become disproportionately more likely to be chosen. In the limiting case where λ → ∞, the strategy with the highest attraction is chosen with probability 1.

### 2.3. Asynchronous updating of repeated-game strategies

The probability that player *i* updates his strategy set in period *t*, 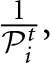 is determined endogenously via the expected length of the block term, 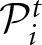, which is updated recursively; that is,^5^


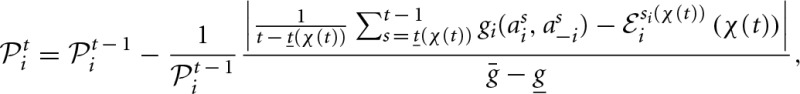


where *t*(χ) is the first period of block χ, and χ(*t*) is the block corresponding to period *t*. In addition, *g* = max_*a*_1_, *a*_2_, *j*_*g*_*j*_(*a*_1_, *a*_2_) is the highest stage-game payoff attainable by either player, and *g* = min_*a*_1_, *a*_2_, *j*_*g_j_*(*a*_1_, *a*_2_) is the lowest stage-game payoff attainable to either player. The normalization by $\frac{1}{\bar{g}-\underline{g}}$ ensures that the expected block length is invariant to affine transformations of the stage-game payoffs. The variable 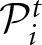 begins with an initial value 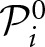. This prior value can be thought of as reflecting pre-game experience, either, due to learning transferred from other games, or due to (publicly) available information. The law of motion of the expected block length depends on the absolute difference between the *actual* average payoff thus far in the block and the *expected* payoff of strategy *s_i_*. The expected payoff for player *i*, 
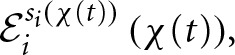
 is the average payoff that player *i* expects (anticipates) to receive during block χ(*t*) and is calculated at the beginning of the block. The difference between actual and expected payoff is thus a proxy for (outcome-based) surprise. As Erev and Haruvy (2013) indicate, surprise triggers change; that is, inertia decreases in the presence of a surprising outcome^6^. In addition, a qualitative control is imposed on the impact of surprise on the expected block length. Multiplying the absolute difference by 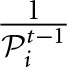 ensures that when the expected block length is long, surprise has a smaller impact on the expected block length than when the expected block length is short.

## 3. Results

**Figure 1:**
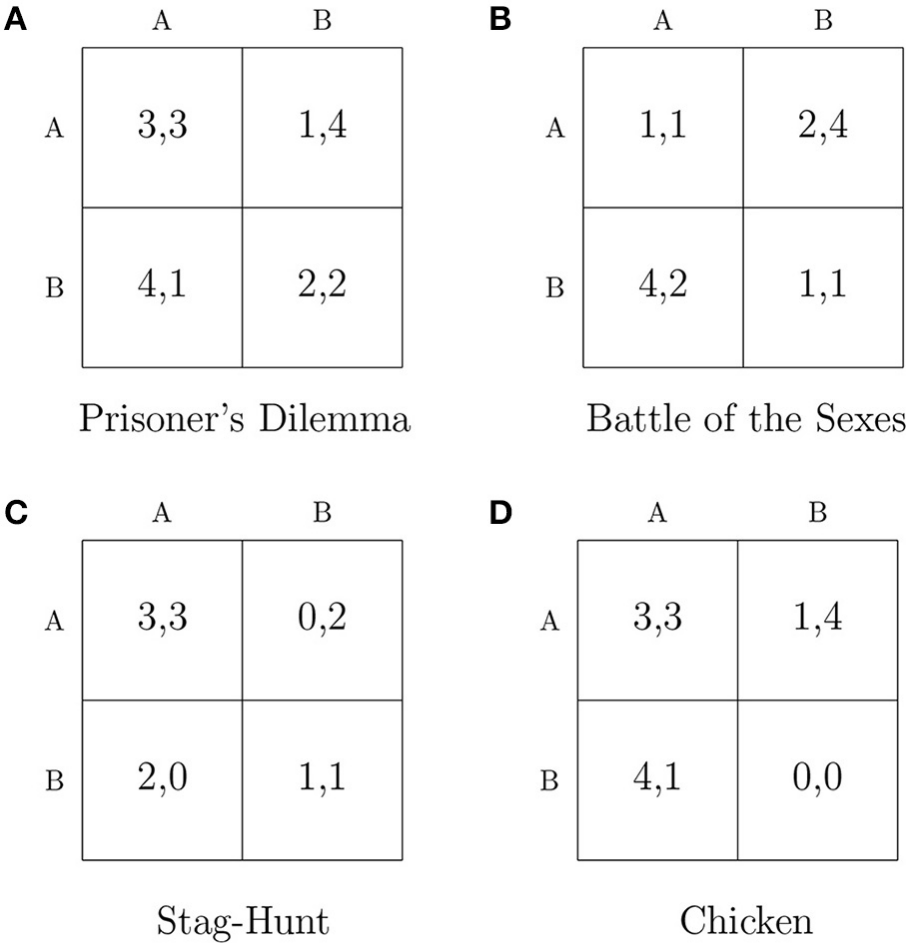
**Payoff Matrices**.

**Figure 2:**
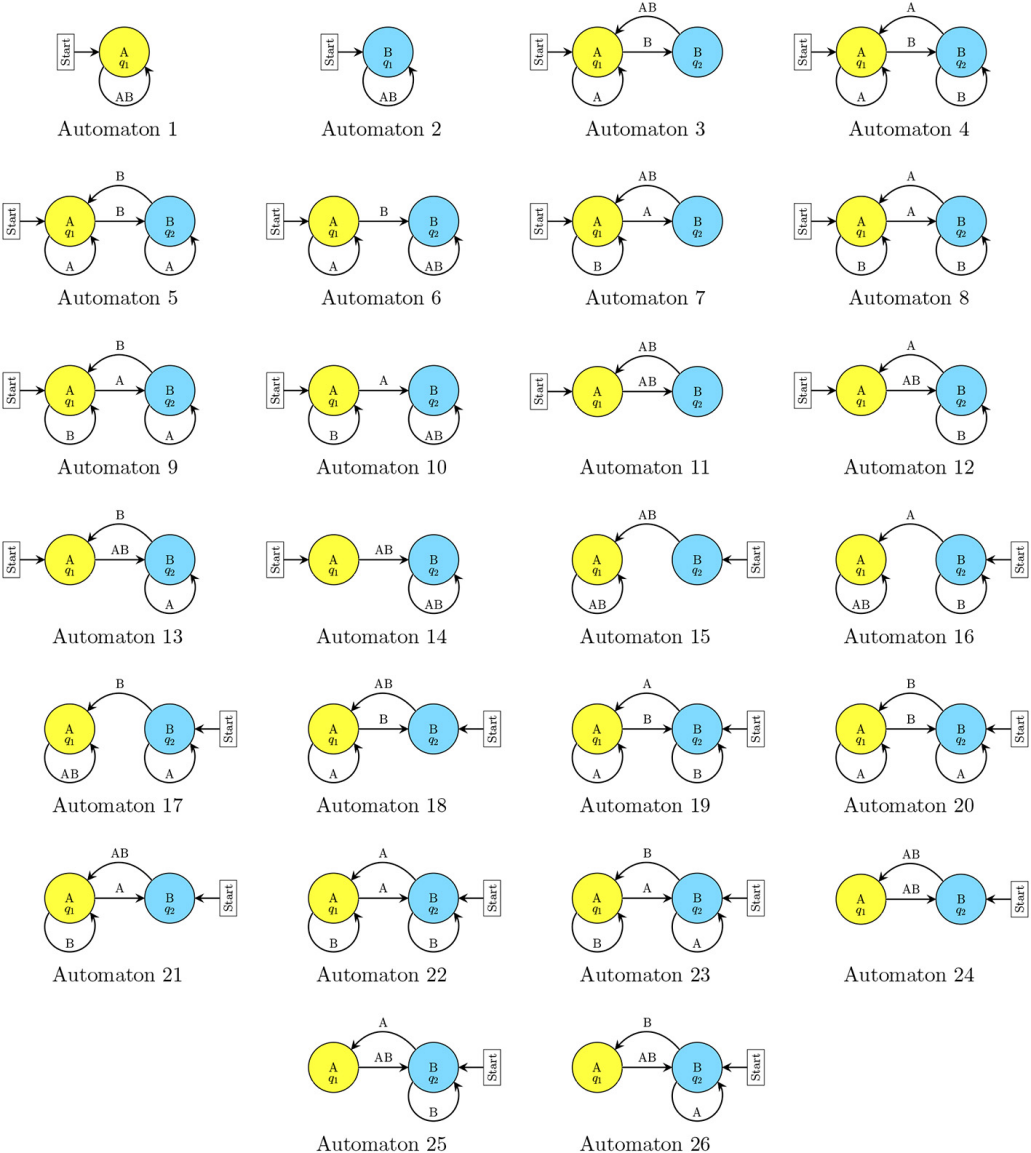
**One-state and two-state automata**.

We study next the relative frequency, speed of convergence and progression of a set of repeated-game strategies in four symmetric 2 × 2 games: Prisoner's Dilemma, Battle of the Sexes, Stag-Hunt, and Chicken. The payoff matrices of the games are illustrated in Figure 1. For the computational simulations, we chose to limit the number of potential strategies considered so as to reflect elements of bounded rationality and complexity as envisioned by Simon (1947). Thus, the players' strategies are implemented by a type of finite automaton called a *Moore machine*. Figure 2 depicts a player's candidate strategy set, which consists of one-state and two-state automata. A formal description is provided in Supplementary Material.

In the simulations, players engage in a lengthy process of learning among strategies. At the beginning of the simulations, each agent is endowed with initial attractions *A^j^_i_*(0) = 1.5 for each strategy *j* in *S_i_*^7^ and initial experience *N_i_* (0) = 1. Players are matched in fixed pairs and update their attractions at the end of each block. The play ends when the average payoff of a given pair converges. More specifically, each simulation is broken up into epochs of 100 periods. The simulation runs until the average epoch payoff of the pair has not changed by more than 0.01 from the previous epoch (in terms of Euclidean distance) in 20 consecutive epochs^8^. The simulations use an intensity parameter λ = 4 in the logit specification^9^. The initial value of 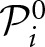 is set to 100 periods^10^. The results displayed in the plots are averages taken over 1000 simulated pairs. At the start of the simulations, each of the strategies in a player's candidate strategy set has an equal attraction and hence an equal probability of being selected. This phase is a lengthy learning process that ends when the average payoff of a given pair of automata converges. We elaborate next on the results of the computational simulations.

### 3.1. Relative frequency and speed of convergence

**Figure 3:**
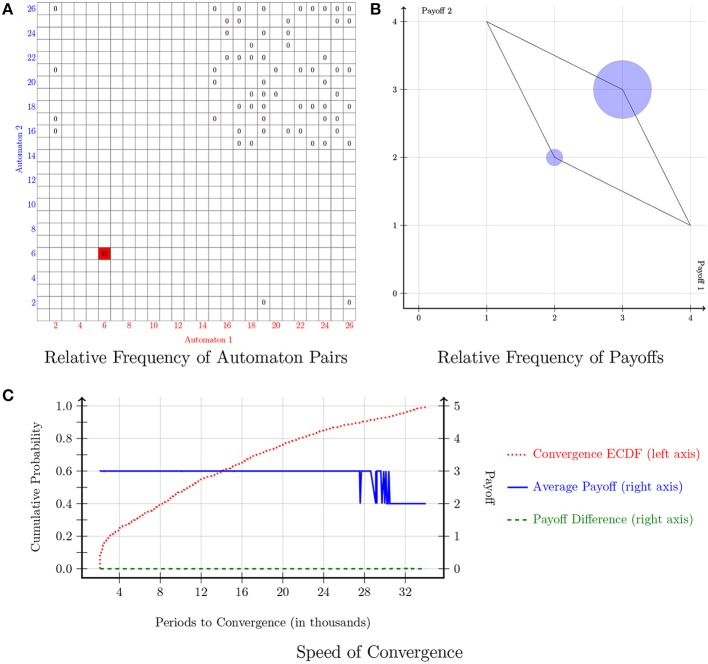
**Prisoner's Dilemma**. Notes: Figures 3–**6** follow the same structure. **(A)** Shows the relative frequency of play across the 26^2^ automaton pairs over the last 1000 periods. The relative frequency of an automaton pair is the number of times the automaton pair occurred normalized by the total number of occurrences of all automaton pairs. The relative frequency of a given pair is denoted by a square located on the coordinates that correspond to that pair; the darker (more red) the square, the higher the (relative) frequency of that pair. In addition, the relative frequency (as a percentage rounded to the nearest integer) is displayed for each pair that appeared at least once in the simulations. If the relative frequency is <0.5% it shows as a 0. **(B)** Shows the relative frequency of each payoff combination over the final 1000 periods and the set of feasible payoffs. The radius of the circle is $r=\frac{\sqrt{RF}}{2}$, where RF is the relative frequency. Note that this is a concave function which emphasizes points with small relative frequency. **(C)** Provides information on the speed of convergence. The left axis indicates the probability and the red dotted line denotes the Empirical Cumulative Distribution Function (ECDF) for convergence. On the other hand, the blue solid line and the green dashed line correspond to the right axis and provide information on the payoffs of the automaton pairs when *averaged over* the last 1000 periods. The blue solid line represents the average payoff of the automaton pair $\left(\frac{g_{1}+g_{2}}{2}\right)$. The green dashed line represents the absolute payoff difference of the automaton pair (|*g*_1_ − *g*_2_|). Points on the blue solid and the green dashed line are sorted according to the corresponding point on the red dotted line.

The payoff matrix of the Prisoner's Dilemma game is indicated in Figure 1A. The cooperative action is denoted with the letter “A,” whereas the action of defection is denoted with the letter “B.” Each player's dominant strategy is to play *B*. Figure 3 displays the results of the simulations in the Prisoner's Dilemma. Figure 3A shows the relative frequency of automaton pairs played over the last 1000 periods. The relative frequency of an automaton pair is the number of times the automaton pair occurred normalized by the total number of occurrences of all automaton pairs. Automaton 6, which implements the “Grim-Trigger” strategy, was the one with the most occurrences. It is important to note that the cooperative outcome (*A, A*) is sustained in a pair consisting of Grim-Trigger automata. This finding is confirmed in Figure 3B, which plots the relative frequency of the payoffs. Crucially, even though the majority of automaton pairs converged to the cooperative payoff (3, 3), there, still, exists a small number of automaton pairs, which chose to defect repeatedly and thus earned a payoff of (2, 2). Finally, the plot in Figure 3C provides information on the speed of convergence. The red dotted line denotes the Empirical Cumulative Distribution Function (ECDF) for convergence. The blue solid line and the green dashed line provide information on the payoffs (right axis) of the automaton pairs when *averaged over* the last 1000 periods. The blue solid line represents the average payoff of the automaton pair $\left(\frac{g_{1}+g_{2}}{2}\right)$. The green dashed line represents the absolute payoff difference of the automaton pair (|*g*_1_ − *g*_2_|). Points on the blue solid and the green dashed line are sorted according to the corresponding point on the red dotted line. About 20% of the simulations converged quite quickly in less than 3000 periods. At this point in time, the blue solid line signifies that the average payoff of the automaton pairs was 3. Given the convergence criterion, we can deduce that about 20% of the automaton pairs started off by cooperating and maintained cooperation until convergence. The next 70% of the simulations were (roughly) uniformly distributed across the range of 3000−27,000 periods. The last 10% of the simulations converged in the range of 27,000−34,000 periods. Looking at the green and blue lines, we observe that the pairs that were converging in less than 27,000 periods ended up at the cooperative outcome, while the pairs that converged at 31,000 periods and beyond converged to the defecting outcome. After 31,000 periods, the automaton pairs that did not attain cooperation experienced short expected block lengths, which prompted them to constantly update the strategies in a manner similar to action-learning models hence converged to the defecting outcome. Pairs that converged between 27,000 and 31,000 periods ended up in either the cooperating or the defecting outcome.

**Figure 4:**
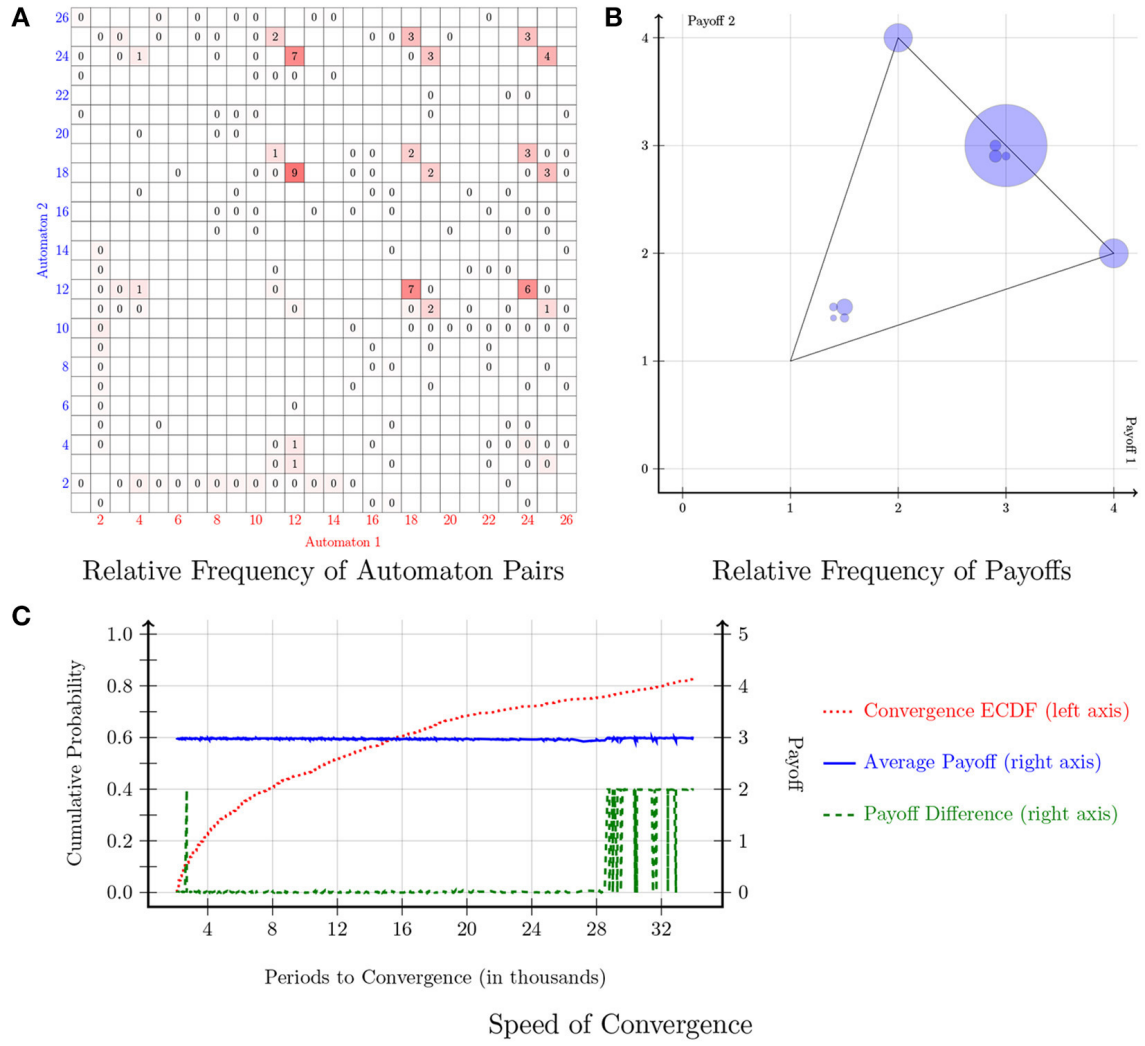
**Battle of the sexes**.

The payoff matrix of the Battle of the Sexes game is indicated in Figure 1B. In this game, there are two pure-strategy equilibria: (*A, B*) and (*B, A*). Figure 4 shows the results of the simulations. In particular, Figure 4A shows the relative frequency of automaton pairs played over the last 1000 periods. The plot covers a large number of automata although Automaton 12 and Automaton 18 show up most frequently. Automaton 12 switches actions every period unless both players choose *B* in the previous period. Automaton 18 switches actions every period unless both players choose *A* in the previous period. Therefore, a pair consisting of Automaton 12 and Automaton 18 would end up alternating between the two pure-strategy Nash equilibria of the stage game. Each automaton would thus earn an average payoff of 3. This is shown in Figure 4B. Arifovic et al. (2006) indicate that standard learning algorithms have limited success in capturing the alternation between the two pure-strategy Nash equilibria in the Battle of the Sexes game. Yet in the proposed model, automata predominantly converge on alternating behavior between the two actions. Finally, a few pairs converged to one of the two pure-strategy Nash equilibria. Figure 4C provides information on the speed of convergence. The automaton pairs can be classified into two groups: (1) those which converged to alternations, and (2) those which converged to one of the pure-strategy Nash equilibria. The pairs that converged to alternations are denoted by the green dashed line at a payoff of 0 (i.e., players within the pairs earned the same payoff). These pairs converged in less than 28,000 periods. On the other hand, the pairs which converged to one of the two pure-strategy Nash equilibria are denoted by the green dashed line at a payoff of 2. The latter pairs took between 28,000 and 34,000 periods to converge.

**Figure 5:**
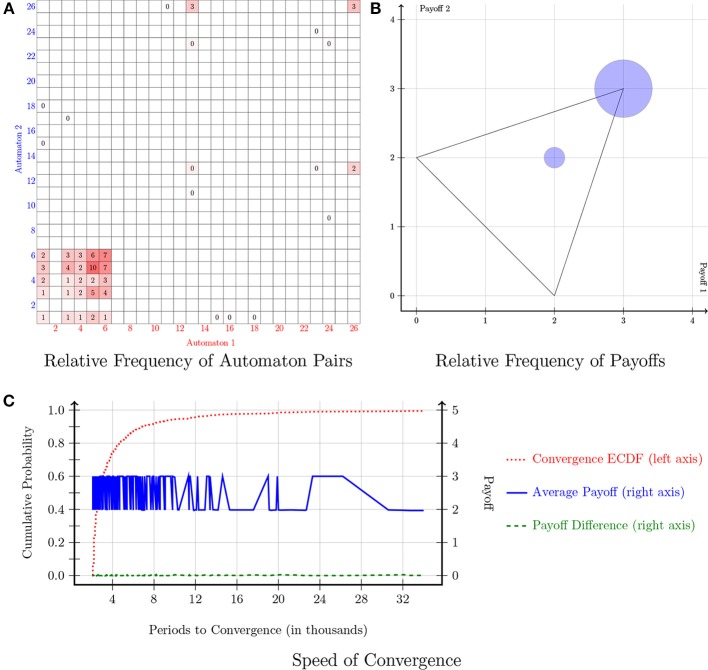
**Stag-hunt**.

The payoff matrix of the Stag-Hunt game is indicated in Figure 1C. In this game, there are two pure-strategy Nash equilibria: (*A, A*) and (*B, B*). However, outcome (*A, A*) is the Pareto dominant equilibrium. Figure 5 shows the results of the simulations. The relative frequency of automaton pairs in Figure 5A suggests that a relatively small set of automata was chosen. Automaton 5, which implements the “Win-Stay, Lose-Shift” strategy, and Automaton 6, which implements the “Grim-Trigger” strategy were the ones with the most occurrences. Other automata that were chosen frequently included: Automaton 1, Automaton 3, Automaton 4, and Automaton 26. It is important to note that with the exception of Automaton 26, any pair combination from this small set of automata yields a payoff of 3 as both players choose (*A, A*) repeatedly. Automaton 26 paired with Automaton 26 corresponds to alternating between the two pure-strategy Nash equilibria, which yields an average payoff of 2. Figure 5B confirms that the most likely outcome is for both players to choose *A* repeatedly. Note that there is also a small number of pairs that converged to (2, 2). Figure 5C shows that convergence in the Stag-Hunt game was quite fast. More specifically, 90% of the pairs converged within only 6000 periods. The blue solid line oscillates mostly between an average payoff of 3 and an average payoff of 2, while the green dashed line indicates that, in either case, the average payoff difference of the automaton pairs was 0.

**Figure 6:**
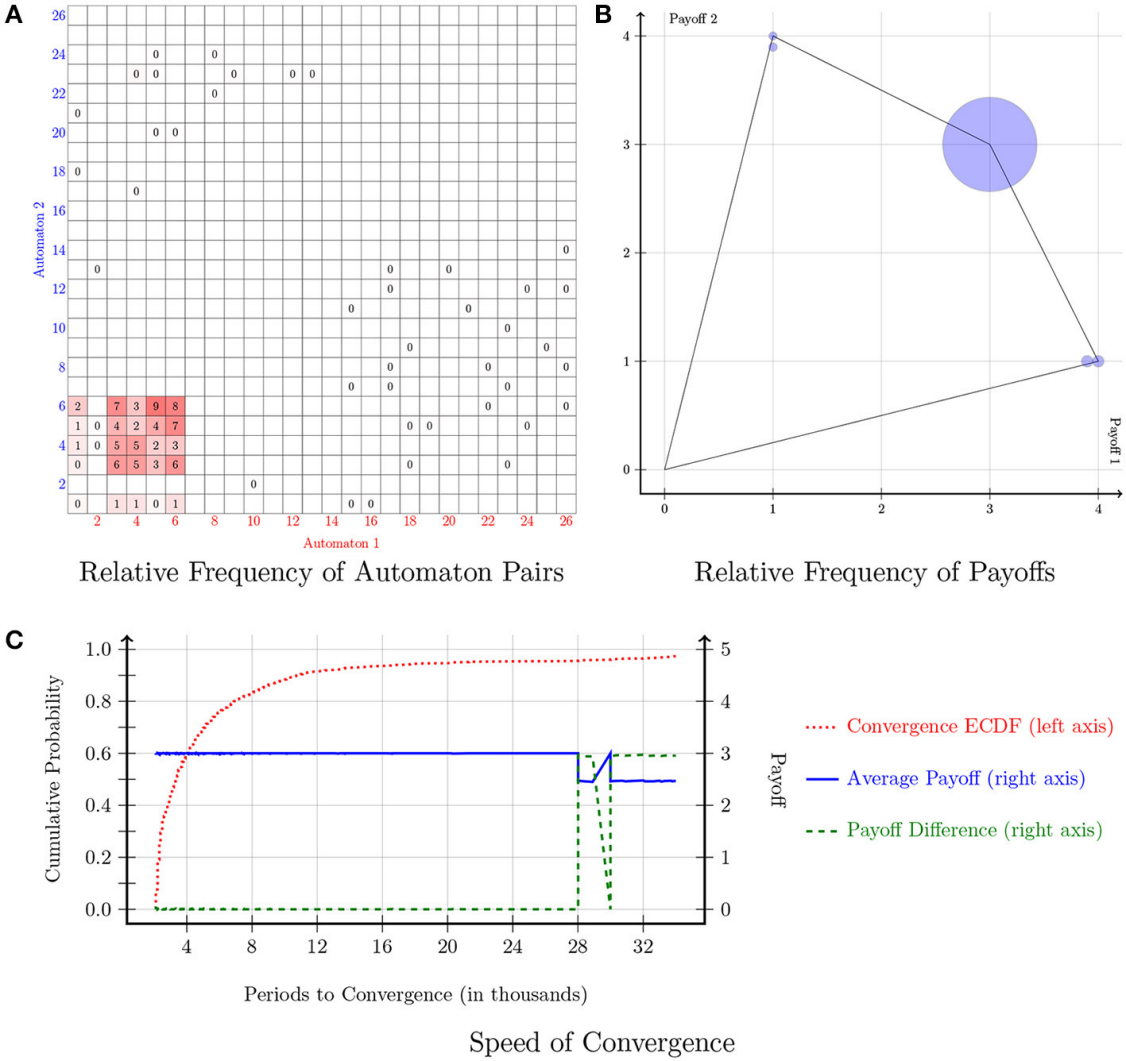
**Chicken**.

The payoff matrix of the Chicken game is indicated in Figure 1D. In this game, there are two pure-strategy Nash equilibria: (*A, B*) and (*B, A*). Recall that in the Chicken game, the mutual conciliation outcome of (*A, A*) yields higher payoffs than the average payoffs for each of the players when alternating between the pure-strategy Nash equilibria. Figure 6 shows the results of the simulations. The results in plots (a) and (b) confirm that game-play converged to a small set of automata: Automaton 3, Automaton 4, which implements the “Tit-For-Tat” strategy, Automaton 5, which implements the “Win-Stay, Lose-Shift” strategy, and Automaton 6, which implements the “Grim-Trigger” strategy. In addition, a very small number of pairs converged to one of the two pure-strategy Nash equilibria. The simulations work in a similar manner to those in the Prisoner's Dilemma game. The automaton pairs, which converged quickly to the conciliation outcome are those that started off by conciliating. Some other automaton pairs that did not establish conciliation from the beginning managed eventually to attain the conciliation outcome. Finally, the rest ended up in one of the two pure-strategy Nash equilibria. The latter observation is evident by the blue line, which indicates an average payoff of 2.5 for the pairs that converged toward the end.

In summary, the extension of the self-tuning EWA model from actions to a simple class of repeated-game strategies improves predictions in two distinct ways. First, it allows for convergence to non-trivial sequences, such as alternation in the Battle of the Sexes game. Second, the richer set of strategies allows the emergence of sophisticated strategic behavior, which not only incorporates punishments and triggers, but also *anticipation* of punishments and triggers. Such sophisticated behavior is instrumental in capturing cooperative behavior in the Prisoner's Dilemma game and mutual conciliation in the Chicken game, precisely, because the threat of punishment may drive a selfish player to conform to cooperation and conciliation in the two games. An alternative approach could be to assume a mixture of adaptive and sophisticated players. An adaptive player responds to either the payoffs earned or the history of play, but does not anticipate how others are learning, whereas a sophisticated player responds to his forecasts using a more sophisticated forward-looking expected payoff function and a mental model of an opponent's behavior (see Camerer et al., 2002; Chong et al., 2006; Hyndman et al., 2009, 2012). Yet such teaching models' inability to both execute *and* anticipate sophisticated behaviors, impedes the delivery of cooperation and conciliation in the Prisoner's Dilemma game and the Chicken game, respectively. Take, for instance, learning in the Prisoner's Dilemma game. Assume that there exists a population of agents, which consists of sophisticated players and adaptive players á la Camerer et al. (2002). An adaptive player always chooses to defect, regardless of his belief about the opponent's action, because defection is a strictly dominant action. On the other hand, a sophisticated player is able to anticipate the effect of his own behavior on his opponent's actions. However, this is not sufficient to drive a sophisticated player paired with an adaptive player to cooperative behavior because the adaptive player will choose to defect, as defection is always his best response. Consequently, the sophisticated player will also respond with defection, and, thus, the pair will lock themselves into an endless string of defections. Analogous arguments hold for the Chicken game; that is, a teaching model with sophisticated and adaptive players would predict the Nash equilibrium-not, the mutual conciliation outcome.

### 3.2. Progression

**Figure 7:**
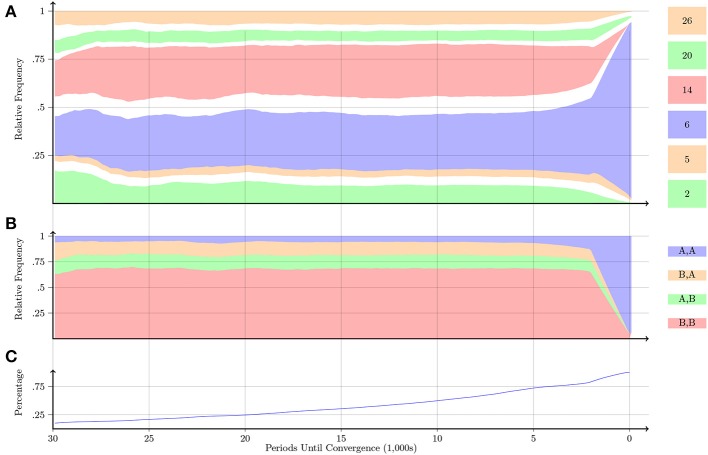
**Prisoner's Dilemma**. Notes: Figures 7–**10** follow the same structure. The plots display information about the progression of play relative to the periods until convergence. The far right of all plots (labeled as “0” on the x-axis) is the point of convergence. **(A)** Shows the progression of the relative frequency of play across the 26 automata over the last 30,000 periods. The automata are ordered starting from Automaton 1 and moving up to Automaton 26. The height of a region at a certain x-value denotes the relative frequency with which an automaton was played at a given number of periods before convergence. We display in color only those automata with a relative frequency of at least 10% in the 30,000 periods before convergence; the remaining automata are represented by the white regions. **(B)** Shows the progression of play of each of the four action profiles. **(C)** Displays the percentage of pairs that took longer than the given x-value to converge. For example, we observe in **(C)** that roughly 25% of the pairs took more than 20,000 periods to converge (and 75% of the pairs took less than 20,000 periods to converge). Thus, the corresponding x-values in **(A)** and **(B)** only reflect 25% of the pairs. All plots are smoothed by taking the average over the previous 2000 periods of play.

**Figure 8:**
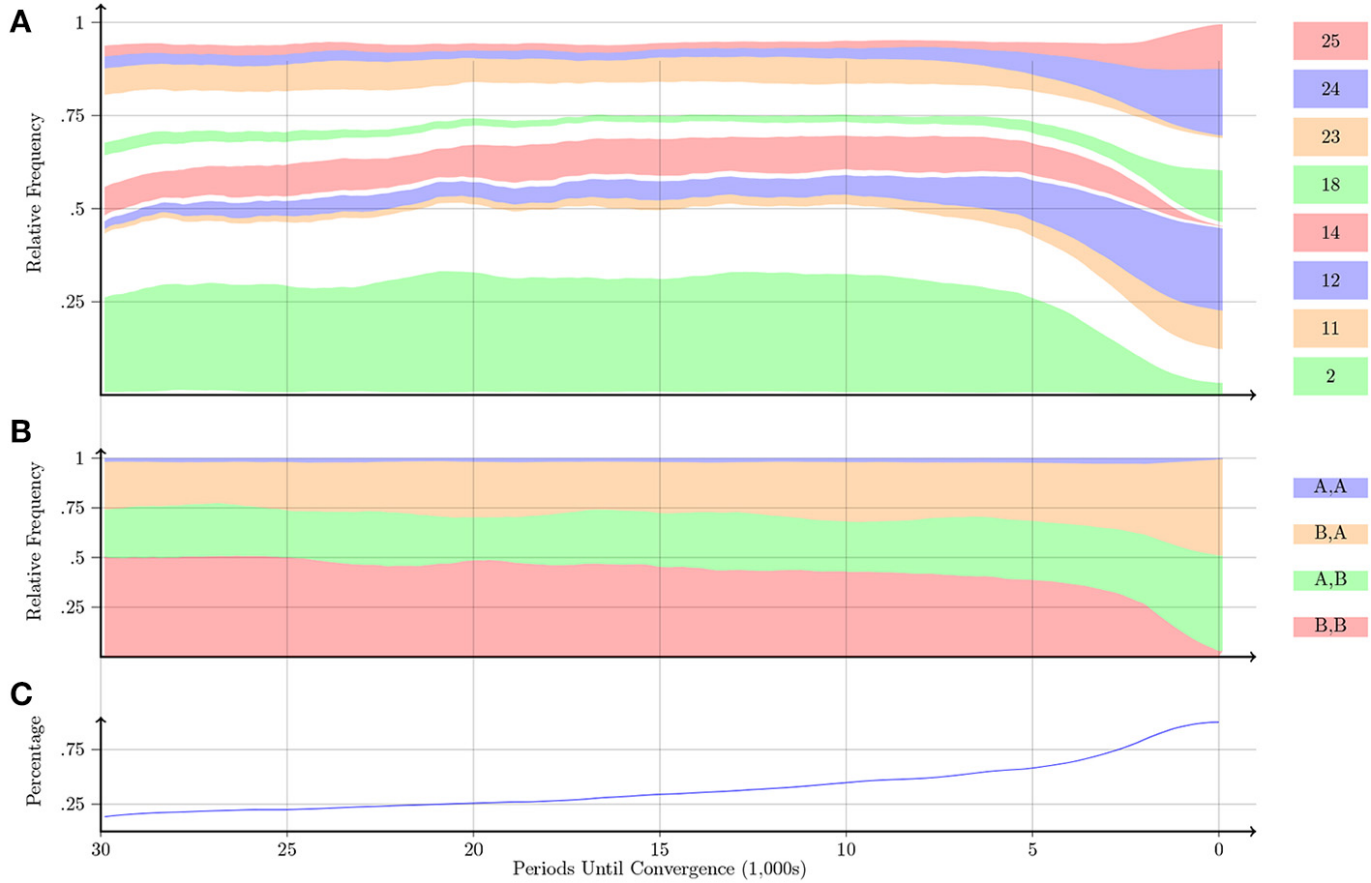
**Battle of the Sexes**.

**Figure 9:**
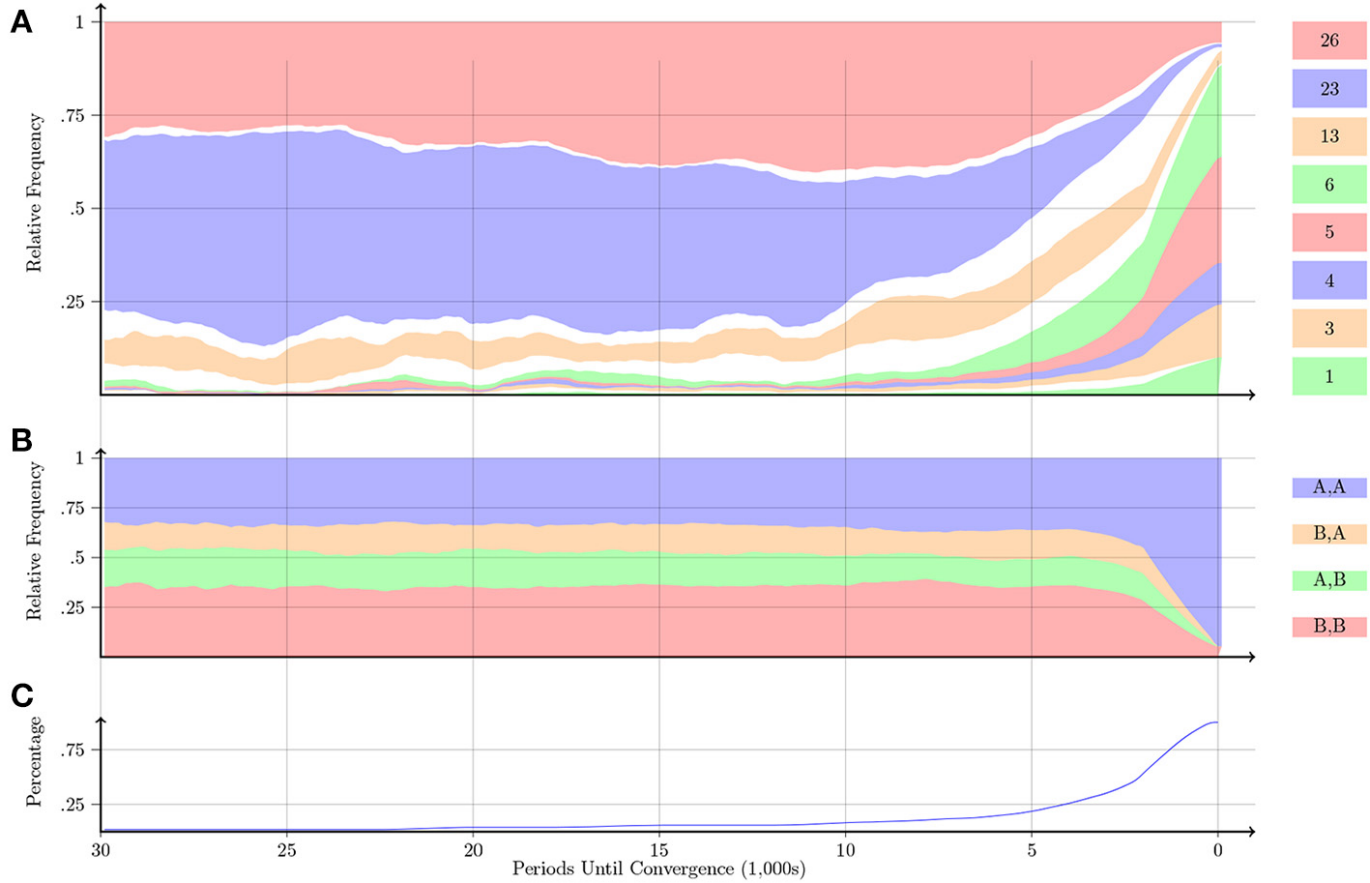
**Stag hunt**.

**Figure 10:**
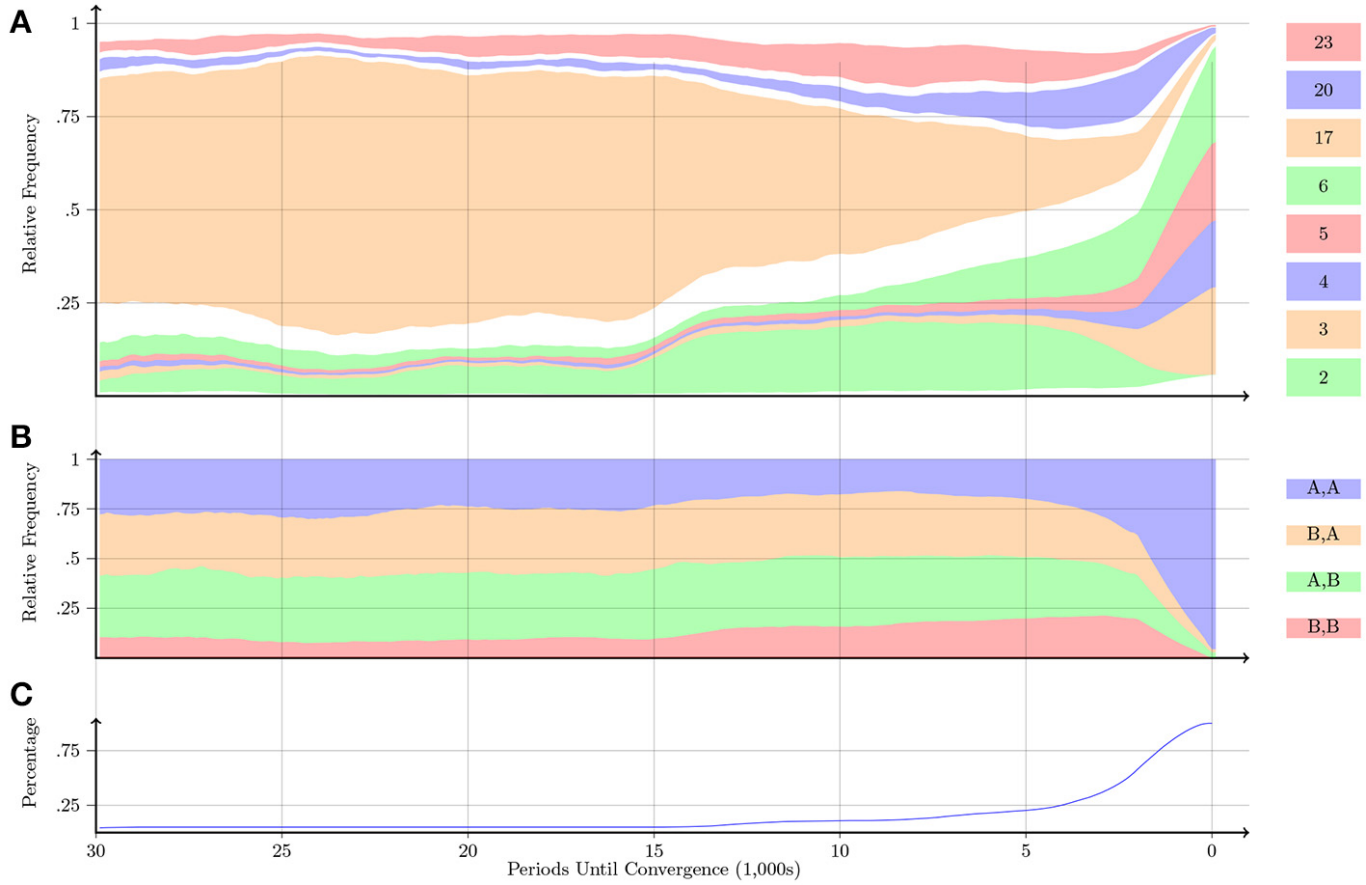
**Chicken**.

Figures 7–**10** display information about the progression of play relative to the periods until convergence for the Prisoner's Dilemma, Battle of the Sexes, Stag-Hunt and Chicken games, respectively. Figure 7 displays information about the progression of play for the Prisoner's Dilemma game. Figure 7A confirms that Automaton 6, which implements the “Grim-Trigger” strategy, was the one with the most occurrences. In the same panel, we also observe that in the earlier periods, Automaton 14 was played almost as frequently as Automaton 6. Automaton 14 plays *A* one time, and plays *B* from then on. Automaton 14 is gradually phased out. Figure 7B indicates that pairs are playing the uncooperative outcome (*B, B*) around 70% of the time before convergence; eventually, the pairs learn to play the cooperative outcome (*A, A*).

Figure 8A shows that in the Battle of the Sexes game, the pairs that take a long time to converge predominately play the preferred action *B*. Eventually pairs learn to play automata that alternate between the two pure-strategy Nash equilibria. Figure 8B shows that 5000 periods before convergence about half of the time pairs are playing the non-equilibrium outcome (*B, B*) and half of the time pairs are playing one of the two pure-strategy Nash equilibria. Pairs rarely ever play the (*A, A*) outcome^11^. Eventually pairs either play one of the two pure-strategy Nash equilibria or alternate between the two pure-strategy Nash equilibria. Furthermore, by the time convergence is reached, only a small percentage of pairs are stuck in an inefficient war-of-attrition outcome.

Figure 9A shows that in the Stag-Hunt game, the small percentage of pairs that took more than 5000 periods to converge predominately play automata which alternate between the two pure-strategy Nash equilibria. This is confirmed in plot (b) as the frequency of the two Nash equilibria is roughly the same. However, this is only a small percentage of the data since about 80% of the pairs converged quickly in less than 5000 periods. Those pairs that converge quickly appear to pick one of the cooperative automata (1, 3, 4, 5, 6) from the beginning, which leads to the Pareto-dominant Nash equilibrium.

Figure 10A shows that in the Chicken game, the pairs that took a long time to converge overwhelmingly select Automaton 17. This automaton starts off by playing the preferred action *B*. It continues to do so as long as the co-player plays *A*; otherwise, it switches to *A*. A pair of such automata are quite infrequent, whereas the relative frequency of the other three action profiles is about the same. This is what is observed in Figure 10B. However, analogous to the Stag-Hunt game, the majority of pairs converge to the cooperative outcome in less than 5000 periods and quickly learn to play one of the cooperative automata.

## 4. Conclusion

Recently, Rabin (2013) proposed a research program that called for the portable extension of existing models with modifications that would improve the models' psychological realism and economic relevance. In Ioannou and Romero (2014), we applied this program of research by building on three leading action-learning models to facilitate their operability with repeated-game strategies. The three modified models approximated subjects' behavior substantially better than their respective models with action learning. The best performer in that study was the self-tuning EWA model with repeated-game strategies, which captured significantly well the prevalent outcomes in the experimental data. In this study, we use the model as a computer testbed to study more closely the relative frequency, speed of convergence and progression of a set of repeated-game strategies in four symmetric 2 × 2 games: Prisoner's Dilemma, Battle of the Sexes, Stag-Hunt, and Chicken. In the Prisoner's Dilemma game, the strategy with the most occurrences was the “Grim-Trigger.” In the Battle of the Sexes game, a cooperative pair that alternates between the two pure-strategy Nash equilibria emerged as the one with the most occurrences. Furthermore, cooperative strategies, such as the “Grim-Trigger” strategy and the “Win-Stay, Lose-Shift” strategy, had the most occurrences in the computational simulations of the Stag-Hunt and Chicken games. Finally, we find that the pairs which converged quickly ended up at the cooperative outcomes. On the other hand, the pairs that were extremely slow to reach convergence ended up at non-cooperative outcomes.

Recently, Dal Bó and Fréchette (2013) required subjects to directly design a repeated-game strategy to be deployed in lieu of themselves in the infinitely-repeated Prisoner's Dilemma game. Dal Bó and Fréchette find that subjects choose common cooperative repeated-game strategies, such as the “Tit-For-Tat” strategy and the “Grim-Trigger” strategy. The “Grim-Trigger” strategy is also predicted in the simulations of the Prisoner's Dilemma game. We hope that in the near future similar studies will be carried across other symmetric 2 × 2 games to confirm the ability of the self-tuning EWA model with repeated-game strategies to capture well subjects' behavior in the laboratory. Finally, it would be interesting to determine the influence of small errors on repeated-game strategies. Currently, the only stochasticity of the model enters through the logit decision rule in the early periods before repeated-game strategies accumulate high attractions, which result in near deterministic strategy choice. We know from the received literature (Miller, 1996; Imhof et al., 2007; Fudenberg et al., 2012; Ioannou, 2013, 2014) that the likelihood and type of errors can affect the degree of cooperation and the prevailing strategies. Thus, a fruitful direction for future research would be to test the susceptibility of the results to small amounts of perception and/or implementation errors.

### Conflict of interest statement

The authors declare that the research was conducted in the absence of any commercial or financial relationships that could be construed as a potential conflict of interest.

## Footnotes

^1^A first attempt was undertaken in the study of Chong et al. (2006), albeit the model proposed was specific to the structure of Trust and Entry games.

^2^Alternatively, Hanaki et al. (2005) develop a model of learning of repeated-game strategies with standard reinforcement. Reinforcement learning responds only to payoffs obtained by strategies chosen by the player and, thus, evades the inference problem highlighted above. Yet reinforcement models are most sensible when players do not know the foregone payoffs of unchosen strategies. Several studies show that providing foregone payoff information affects learning, which suggests that players do not simply reinforce chosen strategies (see Mookherjee and Sopher, 1994; Rapoport and Erev, 1998; Camerer and Ho, 1999; Costa-Gomes et al., 2001; Nyarko and Schotter, 2002; Van Huyck et al., 2007).

^3^Traditionally, action-learning models require that the updating of a player's action set occurs at the end of each period. Instead, the proposed methodology in Ioannou and Romero (2014) requires that the updating of repeated-game strategies occurs with the completion of a *block of periods*, where a block typically consists of more than 1 period. Furthermore, players' blocks of periods vary in length and end at different time-periods (see also Section 2.3).

^4^In the context of finite automata (a formal description is provided in Supplementary Material), let *h^t^_i_*(χ) be player *i*'s action in the *t*th period of block χ, and *s*_−*i*_ = (*Q*_−*i*_, *q*^0^_−*i*_, *f*_−*i*_, τ_−*i*_) be a potential automaton for player −*i*. We say automaton *s*_−*i*_ is consistent with *h*(χ) for the last *t*′ periods, if according to the history, it is possible that the other player played automaton *s*_−*i*_ in the last *t*′ periods and, given player *i*'s most recent action, the proposed automaton is in the starting state. Formally, automaton *s*_−*i*_ is consistent with *h*(χ) for the last *t*′ periods if there exists some state *q^t^* ∈ *Q*_−*i*_ such that *h*^*t*^_−*i*_ (χ) = *f*_−*i*_(*q^t^*) and *q*^*t*+1^ = τ_−*i*_(*q^t^, h^t^_i_*(χ)) for all *T_i_*(χ) − *t*′ + 1 ≤ *t* ≤ *T_i_* (χ) and *q*^*T*_*i*_(χ)+1^ = *q*^0^.

^5^For the interested reader, a detailed exposition to asynchronous updating of repeated-game strategies can be found in Ioannou and Romero (2014).

^6^This gap-based abstraction can be justified from the observation that the activity of certain dopamine-related neurons is correlated with the difference between the expected and actual outcomes (see Caplin and Dean, 2007).

^7^The values of the initial attractions are derived from the Cognitive Hierarchy (CH) model of Camerer et al. (2004).

^8^The maximum length in each of the simulation runs was set to 100,000 periods. The average payoff of the pair converged in 1000 × 4 − 47 = 3953 out of the 4000 total simulations. In 47 simulations, all in the Battle of the Sexes game, the average payoff of the pair did not converge; players were playing their preferred outcome most of the time, but there was too much noise for the average payoff of the pair to converge. In all other simulations, the average payoff of the pair converged. The median length for convergence was 10,750 periods in the Prisoner's Dilemma game, 11,400 periods in the Battle of the Sexes game, 2750 periods in the Stag-Hunt game, and 3300 periods in the Chicken game. Given the convergence criterion, the minimum length of periods for a simulation run is 20 × 100 = 2, 000 periods. This implies that, for instance in Stag-Hunt, a pair of players who arrives at convergence in a median length of 2750 periods has reached the convergence point after 7.5 strategy-updates (a pair of players arrives at convergence point in 7.5 × 100 = 750 periods, given that  is set to 100).

^9^The calibration is based on a grid search. We consider a simple goodness-of-fit measure to determine how far the predictions of the model are from the experimental data. The dataset used is from Mathevet and Romero (2012). Subjects were instructed that the continuation probability for an additional period was 0.99; this was common knowledge in all experiments conducted. We compare the average payoffs over the last 10 periods of the computational simulations to the average payoffs over the last 10 periods of the experimental data. To calculate the measure, we first discretize the set of possible payoffs by using the following transformation:

$$D\left(\pi \right)=\varepsilon \left\lfloor \frac{\pi }{\varepsilon }\right\rceil ,$$

where π is the payoff, ε is the accuracy of the discretization and *D*(π) denotes the transformed payoff. Note that the symbolic function ⌊ · ⌉ rounds the fraction to the nearest integer. For example, if ε = 0.5, then the payoff pair (π_1_, π_2_) = (2.2, 3.7) would be transformed to (*D*(π_1_), *D*(π_2_)) = (2, 3.5). We then construct a vector consisting of the relative frequency of each of the transformed payoffs given some ε. We do the same for the experimental data. To determine how far the predictions of the model are from the experimental data, we calculate the Euclidean distance between the model's vector and the vector of the experimental data. If the predictions match the experimental data perfectly, then the distance will have a value of 0. The maximum value of distance is $\sqrt{2}$ for each game. This value is attained if only one payoff is predicted by the model, only one payoff is observed in the experiment, and the two payoffs are different. Crucially, for a given discretization parameter ε, we define the *best goodness of fit* model as the one whose parameter value minimizes the sum of Euclidean distances across the four games studied.

^10^An upper bound of 60 periods was set on the fitness function for computational efficiency; that is, a player can use a maximum of 60 periods when formulating beliefs.

^11^There are several reasons why automata favor action *B* over action *A*. First, if the co-player is selecting an action at random (i.e., selects each action with probability 0.5), then one is better off selecting the most preferred choice; that is, action *B*. Second, if one is trying to set a precedent on preferred choice, they may continually select the preferred choice to make the co-player believe that there is no intention to switch to the other action. In such a case, the co-player may eventually concede and start best-responding to the player's preferred action. However, if a pair is unwilling to concede, then, this will lead to a war-of-attrition outcome where the pair repeatedly goes to their preferred choice. Consider, for example, a pair using Automaton 12, which is the most commonly used automaton in the Battle of the Sexes simulation. Recall that Automaton 12 switches actions every period unless both players choose *B* in the previous period. Thus, a pair using Automaton 12 will mostly alternate between the two pure-strategy Nash equilibria, also play a few times the war-of-attrition profile, but will almost never play the (*A, A*) outcome.
