# Supplementary material for: Learning with repeated-game strategies
Source: Front Neurosci. 2014 Jul 30;8:212. doi: 10.3389/fnins.2014.00212 (PMC4115627; doi:10.3389/fnins.2014.00212)
Supplement: Supplementary file 1 [file Presentation1.PDF]

Learning With Repeated-Game Strategies

Appendix

Christos A. Ioannou
University of Southampton
c.ioannou@soton.ac.uk

Julian Romero
Purdue University
jnromero@purdue.edu

Contents

A Finite Automata **2**

A Finite Automata

A finite automaton is a mathematical model of a system with discrete inputs and outputs. The system can be in any one of a finite number of internal configurations or "states." The state of the system summarizes the information concerning past inputs that is needed to determine the behavior of the system on subsequent inputs. The specific type of finite automaton used here is a Moore machine. A *Moore machine* for player i, M_i, in a repeated game $G = (I, \{\mathcal{A}_i\}_{i \in I}, \{g_i\}_{i \in I})$ is a four-tuple $(Q_i,\ q_i^0,\ f_i,\ \tau_i)$ where Q_i is a finite set of internal states of which q_i^0 is specified to be the initial state, $f_i : Q_i \to \mathcal{A}_i$ is an output function that assigns an action to every state, and $\tau_i : Q_i \times \mathcal{A}_{-i} \to Q_i$ is the transition function that assigns a state to every two-tuple of state and other player's action. It is pertinent to note that the transition function depends only on the present state and the other player's action. This formalization fits the natural description of a strategy as i's plan of action in all possible circumstances that are consistent with i's plans. In contrast, the notion of a game-theoretic strategy for i requires the specification of an action for every possible history, including those that are inconsistent with i's plan of action. It is important to highlight that to formulate the game-theoretic notion of a strategy, one would *only* have to construct the transition function so that $\tau_i : Q_i \times \mathcal{A} \to Q_i$, instead of $\tau_i : Q_i \times \mathcal{A}_{-i} \to Q_i$.

In the first period, the state is q_i^0, and the automaton chooses the action $f_i(q_i^0)$. If a_{-i} is the action chosen by the other player in the first period, then the state of i's automaton changes to $\tau_i(q_i^0, a_{-i})$, and in the second period, i chooses the action dictated by f_i in that state. Then, the state changes again according to the transition function given the other agent's action. Thus, whenever the automaton is in some state q, it chooses the action $f_i(q)$, while the transition function τ_i specifies the automaton's transition from q (to a state) in response to the action taken by the other player. For example, the automaton $(Q_i,\ q_i^0,\ f_i,\ \tau_i)$ in Figure 1 carries out the "Grim-Trigger" strategy. In the transition diagram, a vertex denotes the internal state of the automaton with the prescribed agent's action indicated in the center, and the arcs labeled with the action of the other player indicate the transition to the states.

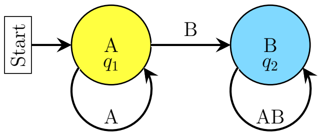

$$Q_i = \{q_1, q_2\}$$
$$q_i^0 = q_1$$
$$f_i(q) = \begin{cases} A & \text{if } q = q_1 \\ B & \text{if } q = q_2 \end{cases}$$
$$\tau_i(q, a_{-i}) = \begin{cases} q_1 & \text{if } (q, a_{-i}) = (q_1, A) \\ q_2 & \text{otherwise} \end{cases}$$

Figure 1: Grim-Trigger Automaton

Notes: The vertices denote the states of the automaton, and the arcs labeled with the action of the other agent indicate the transition to the states.
